# Appendix Extra Limites

**Published:** 1820-09-01

**Authors:** 


### APPENDIX EXTRA LIMITES.

### I.

*Annual Report of the Associated Apothecaries and Surgeon-Apothecaries of England and Wales; held at the Crown and Anchor Tavern, Strand, London, June* 21, 1820, JAMES PARKINSON, Esq. President.

THE Committee has the honour of laying its Annual Report before the General Meeting of the Association.

No particular circumstance has occurred during the past year, demanding immediate exertions, yet has your Committee not been inactive.

The great object which the Association hopes one day to see obtained, namely, a legal enactment, by which the health of the public will be guarded against the manifold evils which daily arise from the practice of uneducated, and, therefore, unqualified persons, has been duly and constantly kept in view.

The Committee is much gratified in being able to state, that the Code of Rules and Regulations, which was sanctioned by the last Annual General Meeting, has been distributed throughout the country; and that District Committees are forming in different parts, with the certainty of their being ultimately very advantageous. In Staffordshire, for instance, not only have the Regulations, recommended for securing the necessary intercourse with the Metropolitan Committee, been carried into effect with zeal and promptitude, but a most beneficial measure has resulted, a Medical and Surgical Library has been established, for the use of the Members of the Association, who reside in that district, of which Wolverhampton may be considered the centre. The Committee hopes to see this excellent mode of inducing medical men thus to associate with each other, acted upon in other districts, as it cannot fail to promote useful discussion, and extend professional knowledge.

The Committee must here express the satisfaction it feels at the great exertions which have been made by the Society of Apothecaries, in carrying into active operation the powers entrusted to them by the Legislature. In the before-mentioned County of Stafford, a resolute stand has been made against empiricism, and the Committee learns with satisfaction, that there are other cases still pending, which will serve to show the energy evinced by the Society of Apothecaries, in protecting the just rights of the legalized Medical Practitioner; in a very short time, these circumstances will become more manifest, as the many obstacles by which the operations of that act have been too frequently impeded and evaded, will be done away.

The rejection of the Surgeons' Act in the late Session of Parliament, at the same time that it must have disgusted the Royal College of Surgeons, by the very unfair, and even unjust manner, in which it was opposed and thrown out, has had the unhappy effect of showing more clearly to irregular practitioners, how little power the Royal College of Surgeons possesses over those who practise surgery in this country.

Under these circumstances, although the practice has been hereby left in a much worse state than before, yet the Association cannot expect that the Royal College of Surgeons will apply to Parliament again for some time to come; and, therefore, the Committee has most anxiously sought for some new channel, through which it may obtain, for society, a proper guarantee, that ignorant persons shall be prevented, by a positive law, from the practice of surgery, and more especially of midwifery; but it has hitherto sought in vain, indeed, as your Committee has been led to conclude, that the desired legal enactments have not been obtained, only because neither the Public nor the Houses of Parliament are yet sufficiently aware of the necessity of the measure, it has of course, for the present, abandoned every idea of recommending this Association to go before Parliament, with a Bill for amending the Practice of Surgery and Midwifery; but has principally turned its thoughts towards the best mode of laying before the Public, such proofs of the evils which necessarily result from the present system, as must render the necessity of Parliamentary influence abundantly manifest.

It is with great pleasure the Committee learns, that "a Series of Letters, addressed to the President of the Associated Apothecaries and Surgeon-Apothecaries of England and Wales, on the present state of the Practice of Physic and Surgery" has been published. The first Series is intended to give a comparative view of particular systems of Medical

Education, to consider the separation of Medicine from Surgery, to estimate the claims of the General Practitioner, and to propose a more respectable mode of remunerating his attendance. The Committee learns this with satisfaction, because it is sure that good will result from laying the present state of Medical Practice before the Public for their consideration in every possible manner.

That the Public are not fully sensible of the varied and precise knowledge which is required before any person can honourably, or even safely practise the art of medicine is perfectly clear, but that the higher classes of Society are even still more uninformed of the gross ignorance which pervades the mass of irregular Practitioners, has been forced upon the notice of the committee by abundant evidence. This is not, however, unnatural, for the higher classes have but little intercourse with medical men, except with Physicians, or with those general Practitioners whose varied and extensive knowledge, and whose medical acquirements constitute them Physicians in every thing but the name.

Urged by these considerations, the Committee has therefore to recommend, that it be an instruction to future Committees to bring the subject into general notice, by all possible means; that the country members be urged to collect, through the medium of the district Committees, such facts as may fairly display the mischief which results from uninformed Practitioners, and may supply a body of evidence ready to be adduced whenever the measure of petitioning is adopted; and especially that, early in the next session, a petition be presented to each House of Parliament, praying its interposition, and stating fully the grounds upon which the Association believes that some legislative enactment is absolutely necessary.

Upon the same principle also, of giving publicity to the claims and the present situation of the General Practitioner, the Committee further recommends the formation of some plan, by which the Members of the Association shall have an opportunity of vindicating their own respectability, and of marking, as perfectly as it may be done, the line between the scientific and the empirical Practitioner.

To do this the more perfectly, the Committee proposes, that an invitation be given to the Members of the Association, and indeed to all other members of the Medical Profession, to transmit to the Committee, for publication, some portions of that knowledge, which must have resulted from their extensive practice and abundant experience. In this way the Committee hopes to be enabled to publish, at short intervals, volumes of Medical and Surgical Transactions, which will demonstrate how well the General Practitioner deserves the legal sanction which he claims for himself and for the public; and to stimulate him to improve himself further in the God-like art of relieving the countless miseries of a sick bed. The Committee feels that these are only silent labours, but it is, at the same time, convinced that the Association will gain its object as certainly and as quickly by such means, as if it endangered the existence of its small funds by taking more apparently active measures before the time arrives, when such measures may be attended with success.

In conclusion, the Committee cheerfully commits itself to the kind consideration of the Association, regretting only, that its ability to protect the community at large, and to benefit its own respected profession is so disproportionate to its wishes and its zeal.

The state of the Funds of the Association being reported by the Treasurers, viz. (See Page 327.)

The following Resolutions were then carried unanimously,

### Resolutions.

*Resolved*, 1. That the Report now read be received and adopted.

*Resolved.*—2. That a book be prepared and printed, containing the present Report, the Rules of this Association, and a List of the Members; which book may be obtained by any member who applies for it to the Secretary in any mode which will not subject the Association to expence.

*Resolved.*—3. That the grateful acknowledgments of this Association be given to James Parkinson, Esq. for the great ability, the indefatigable attention, and unabated zeal, with which he has, during the last three years, conducted himself in the office of President, and that he be requested to continue in that situation.

This Meeting having heard, with unfeigned regret, the intention of James Parkinson, Esq. not to accept the office of President of the Association for the ensuing year, after having filled it for three successive years, in a manner which has called for, and obtained the thanks of this Association, it was

*Resolved.*—4. That Joseph Hayes, Esq. be elected to the office of President of this Association, *vice,* James Parkinson, Esq. resigned.

*Resolved.*—5. That Joseph Hayes and Arthur Tegart, Esq. be requested to accept the best thanks of the Association, for their zealous services as Vice-Presidents during the last year.

*Resolved.*—6. That the thanks of the Association be given to the Treasurers for their past services, and that their continuance in office be requested.

*Resolved.*—7. That the thanks of this Association be given to the General Committee, for their very able Report, and for their great attention to the concerns of the Association.

*Resolved.*—8. That the best thanks of the Association be presented to the Secretary, John Powell Esq. for the readiness and ability with which he has fulfilled the greatly extended duties of his situation during the last year.

*Resolved.*—9. That Arthur Tegart and C. T. Haden, Esqrs. be Vice-Presidents of this Association for the year ensuing.

No regular order having been observed in placing the names of the General Committee, but it being specified in article iv. section 5, that "half of each class shall go out every year by rotation, such members, nevertheless, being eligible for re-election." The Committee, to avoid the difficulty which now presents itself, suggests that such gentlemen shall be fixed upon to go out, as favoured the Committee with their attendance during the last year the least frequently. This proposal having met with the approbation of the meeting, it was

*Resolved.*—10. That the arrangements of the General Committee respecting the formation of Committees for the year ensuing be adopted.

The members of the Apothecaries' Society who have attended least frequently, being—

| | |
|---|---|
| R. S. Wells, Esq. | R. M. Kerrison, Esq. |
| M. Bowman, Esq. | John Cates, Esq. |
| C. Shillito, Esq. | T. Graham, Esq. |

And the members who do not belong to the Apothecaries' Society who have attended least frequently, being—

| | |
|---|---|
| R. Ogle, Esq. | A. T. Thompson, Esq. |
| P. Fernandez, Esq. | Lewis Leese, Esq. |
| R. James, Esq. | T. Rodd, Esq. |

C. T. Haden being elected Vice-President, it was

*Resolved.*—11. That

| | |
|---|---|
| R. S. Wells, Esq. | Edward Brown, Esq. |
| Henry Field, Esq. | Thomas Parrot, Esq. |
| Jos. Hurlock, Esq. | Charles Shillito, Esq. |

belonging to the Apothecaries' Society, and that

| | |
|---|---|
| James Parkinson, Esq. | Nodes Dickenson, Esq. |
| Thomas Alcock, Esq. | Robert James, Esq. |
| George R. Rodd, Esq. | Lewis Leese, Esq. |

Thomas Morrah, Esq.

Non-members of the Apothecaries' Society, be elected to fill the vacancies of the General Committee for the year ensuing.

*Resolved.*—12. That the day of the Annual Meeting and Anniversary Dinner be changed to the first Wednesday in July; the hour of meeting to be *three* o'clock instead of *two;* and that of the *dinner* to be *six* o'clock, instead of *five*.

*Resolved.*—13. That the General Meeting most earnestly recommend to the members of this Association who reside in the country, the imitation of the Staffordshire District Committees, in the establishment of Medical and Surgical Libraries, in such central situations as may appear most likely to be beneficial to the members resident in such districts.

*Resolved.*—14. That the General Meeting feel themselves called upon to express very strongly the grateful sense they entertain of the benefits which have resulted to the public, and to the medical profession, from the zealous and active exertions that have been made by the Society of Apothecaries, in carrying the Apothecaries' Act into efficient operation.

*Resolved.*—15. That the General Meeting do recommend to the General Committee, to take into their consideration, at a future Meeting, the present mode employed by the Chemists and Druggists, of acting as Apothecaries, and even visiting patients, under the supposed sanction of Clause 28, *(vid. Apothecaries' Act)* in opposition both to the spirit and principle of that Act, passed in the year 1815, for the regulation of the Practice of Apothecaries, &c. &c.

*Resolved.*—16. That the attention of the Committee be directed to the propriety of presenting a Petition to each House of Parliament, to take into their most serious consideration the deplorable manner in which the health and lives of the community are hazarded, by the want of a proper legal control over illiterate and unqualified persons practising Surgery and Midwifery.

*Resolved.*—17. That the members resident in the country be urged to collect, through the medium of District Committees, such facts as may more fully display the mischiefs which result from the practice of uninformed and ignorant persons, and may supply a body of evidence, ready to be adduced whenever a new application shall be made to Parliament.

*Resolved.*—18. That the Committee be directed to take the necessary measures for inviting the members of the Association, and all other members of the Medical Profession, to transmit to them papers on medical and surgical subjects, for publication, in occasional volumes, to be entitled, "Transactions of the Associated Apothecaries and Surgeon-Apothecaries of England and Wales;" or such other title as the Committee may hereafter approve.

*Resolved.*—19. That the Editors of the London Medical Repository, and London Medico-Chirurgical Review, be respectfully requested to insert the Report, &c. now adopted, in their respectful valuable publications.

*Resolved.*—20. That the General Committee be directed to lay the objects of the Association before the public in all possible ways.

JOSEPH HAYES, *President.*

June 21, 1820.

---

### FUNDS.

*Statement of Account, from July* 21, 1819, *to June* 21, 1820.

| | *Dr.* | £. | *s.* | *d.* |
|---|---|---|---|---|
| 1819. | | | | |
| July 23. | To Mr. Powell, the Secretary, for Sundries and Salary to June 24, 1819 - - - - - - | 37 | 6 | 0 |
| Dec. 20. | For Advertisements - - - - - - - - - - - - - - | 16 | 19 | 6 |
| | Sundries to Mr. Parkinson, audited July 21, 1819, but not drawn - - - - - - - - - - - | 3 | 12 | 6 |
| 1820. | | | | |
| June 21. | To Mr. Powell, for Sundries and Advertisements to June 21, 1820 - - - - - - - - - | 13 | 0 | 3 |
| | | £. 70 | 18 | 3 |

| | *Cr.* | £. | *s.* | *d.* |
|---|---|---|---|---|
| 1819. | | | | |
| | By Balance in the Bankers' Hands | 124 | 6 | 0 |
| 1820. | | | | |
| Feb. 19. | Subscriptions through J. Upton, Esq. - - - - | 23 | 2 | 0 |
| June 14. | One Year's Dividend of £.500 3 per Cents. | 15 | 0 | 0 |
| June 21. | Subscriptions through Mr. Powell - - - - - - | 3 | 7 | 0 |
| | | 170 | 15 | 0 |
| | | 70 | 18 | 3 |
| | Balance in Bankers' Hands | £. 99 | 16 | 9 |

3 per Cents. £. 500.

We have audited this Account and find it correct, June 21, 1820.

C. T. HADEN,

E. LEESE.

*Officers of the Associated Apothecaries and Surgeon-Apothecaries, &c. for the Year* 1820.

PRESIDENT.—JOSEPH HAYES, Esq.

VICE-PRESIDENTS.—ARTHUR TEGART, Esq.; C. T. HADEN, Esq.

Treasurers.—JAMES PARKINSON, Esq.; JAMES UPTON, Esq.; JOHN HUNTER, Esq.

### *GENERAL COMMITTEE.*

*Members who belong to the Society of Apothecaries.*

James Upton, Esq.; John Hunter, Esq.; James Seaton, Esq.; Walter Drew, Esq.; Wentworth Malim, Esq.; William Hillman, Esq.; R. S. Wells, Esq.; Henry Field, Esq.; Edward Browne, Esq.; Joseph Hurlock, Esq.; Charles Shiletto, Esq.; John Parrott, Esq.

*Members who do not belong to the Society of Apothecaries.*

William Acret, Esq.; George Fincham, Esq.; Thomas Hurst, Esq.; Neville Wells, Esq.; Edward Leese, Esq.; Lewis Leese, Esq.; James Parkinson, Esq.; Nodes Dickenson, Esq.; Thomas Alcock, Esq.; George R. Rodd, Esq.; R. James, Esq.; Thomas Morrah, Esq.

Bankers.—Messrs. GOSLING and SHARP, Fleet Street.

Secretary.—JOHN POWELL, 62, Newman Street.

We are glad to see a proposition made by the Associated General Practitioners for the publication of Periodical Transactions, after the example of other Societies. No body of men can possibly have the command of such ample materials for the construction of a work of this kind, as the Associated Apothecaries and Surgeon-Apothecaries of Great Britain. Their medical education is now perfectly adequate to the task of observing scientifically, and recording accurately, the phenomena of disease, the operation of remedies, and the disorganizations of structure, which come within the sphere of their practice. And as this class of the profession form the great numerical majority of it, and possess the exclusive opportunities of seeing daily the whole course of diseases, and the exact effects of the medicines employed, so they are, in reality, the best qualified to collect the materials of our knowledge, and advance the progress of our science. The publication of an annual, or semi-annual volume of

Transactions, with a list of those who are legally qualified to practise, would not only make the members of this extensive body more known to each other, and to the public; but would constantly generate and cherish a zeal in the prosecution of medical investigations, and an increased attention to the phenomena of Nature, whether in health or disease. We think it highly probable, too, that literary and scientific enterprizes of this kind, would have a strong tendency to harmonize the profession, inspire liberality of sentiment, check the selfish passions, and dissolve that Odium Medicum, which too often operates with such baleful influence on an otherwise enlightened order of society.

We shall here take the liberty of throwing out a few suggestions for the consideration of the Committee, on this subject.

As it is highly probable that materials for an annual volume, at least, will be readily supplied, as soon as the design is fully made known, we conceive, that economy might be insured, and the possibility of risk avoided, by some such plan as the following:—We would recommend, that Subscribers be solicited, on the condition that the work be delivered to them at prime cost, or a very little above that sum. Thus suppose seven hundred subscribe, and one thousand copies of an octavo volume, of four hundred pages, be printed. The paper and printing of the volume, if properly managed, and no plates introduced, should not cost more than one hundred and thirty pounds; and allowing that this sum, together with the expence of boarding the work, be laid upon the seven hundred Subscribers, the price of each volume will be about four shillings and sixpence, which is not more than half the sum they would otherwise pay for a similar volume. There would then be three hundred copies left for the public demand, the money resulting from which might be appropriated as was thought most advisable by the Committee.

---

### II.

*Observations on the relative Powers of the Heart and Vessels in the Support of the Circulation of the Blood.* By C. E. Lucas, M. D. of Hatfield.

The fact of the pulse not being accompanied by any general dilatation of the arteries, in the ordinary state of the circulation, may, I hope, now be taken for granted; as the evidence adduced in Dr. Parry's work on the Arterial Pulse, with that of others referred to in your article on this subject, at page 201, vol. ii. of your Journal, is so conclusive, that as you say, "it is hardly credible that any man can continue to support the old doctrine, (of alternate dilatation and contraction) who is at all open to the evidence of truth."

It must be, I think, equally clear, that if the blood projected into the aorta by the systole of the heart, dilated that vessel altogether, it could not be propelled forward; for certainly, if the dilatation were equal to the projected quantity, there could be no propulsion, until, by the subsequent contraction, the artery had recovered its usual diameter, when an equal quantity would be displaced; thus the blood would be in alternate motion and rest, and the same effect would be produced upon the circulation, as if a directly contrary mechanism were employed, and the blood were driven by the successive shocks of the heart through tubes incapable of motion. That this is contrary to the fact, we have, I think, indubitable evidence in the case of divided arteries, where the blood is projected in a stream, though alternately to greater distances during the systole of the heart. On the other hand, the denial of *all* dilatation of the aorta consequent to the impulse of the heart, leads inevitably to the same conclusion, of alternate motion and rest of the blood; and Dr. C. Parry is forced to confess, that your reasoning in support of this opinion, "is so consequent," that he "can find no reply but in the opposite matter of fact, and in the supposition that the data themselves are erroneous." This supposition will, I have no doubt, be found true, and to remove the difficulty, it will be only necessary to admit, that the root of the aorta does undergo a *partial* dilatation; as thus, suppose that two ounces of blood are thrust into the aorta at each systole of the heart, and that one ounce and a half of this be propelled along the vessel, but that the root of the aorta, yielding in part to the distending force, may be dilated in its diameter to the extent of the remaining half ounce. During the diastole of the ventricle, the artery will contract upon this blood, and force it necessarily both ways, thereby at once closing the valves, and continuing the progressive motion of the column before, though with diminished impulse, until the vessel has regained its usual diameter, when the systole of the heart will occur.

Dr. Parry himself does not deny that this may take place, expressly saying—

"With regard to the expansion of the root of the aorta, I have already admitted, in a former work, that I have nothing to say in point of fact." And then adds, " it should seem that the muscular fibres which thinly supply that part, (but which he denies to the other parts of the arteries) are intended as a provision against somewhat of that kind, which may, at least, casually occur. If, however, this be admitted, it is no argument for a similar change in other parts of the system."

We know that during the contraction of the right auricle, the venæ cavae suffer a dilatation equal to the temporary pressure of blood upon them; may we not therefore conclude, that a similar state of the root of the aorta takes place, from the same cause, under the unyielding state of the arteries generally, thereby giving effect to the valvular apparatus there placed, and providing effectually for the support of a continued flow of the blood. But although the root of the aorta may yield to the distending forces, before stated, the parts beyond it will suffer no dilatation; for in vessels, the aggregate of whose areas is constantly enlarging, and where the momentum must of course be diminishing with the declining velocity, in the same proportion, a moderate resistance of their coats will so far counteract the force of the heart, as to prevent all dilatation. But even the alternation of impulse derived from the heart, will also finally disappear in the minute ramifications of the capillaries, from the resistance of vessels and their numerous anastomoses, the increase of their collective areas, and the demand made upon their circulating fluids by the various secretions and excretions; thus converting the arterial current into a smooth continuous stream. If, therefore, we find all inequality of impulse removed from the blood, before it is applied to its final purposes in the system; must we not, à fortiori, conclude, that a state of alternate motion and quiescence, would be wholly incompatible with the due performance of those functions?

Without some provision for a continued flow of the blood, independent of the heart, the most disastrous results must follow. Any suspension of the action of the heart, would be followed by that of the circulation; and by the removal of the power by which the blood is gradually accumulated on the right side of the heart, the most powerful stimulus to the renewal of its action would be lost; and thus a temporary suspension would terminate in a final extinction of its action. The balance of the arterial and venous systems, the loss of which, under disease, has been so beautifully illustrated by Dr. Armstrong, could not be maintained; indeed, every important function of the arteries, both in health and disease, seems connected with their possessing an independent power of action. These observations will not apply so much to those supposed cases of alternate motion and quiescence of the blood, dependant on alternate dilatation and contraction of the arteries, because, here, the contraction of the vessels might be supposed to be continued, after the dilatation from the returning systole of the heart had ceased; but where, on the contrary, the blood is supposed to be quiescent in the vessels during the diastole of the ventricle, it does not appear how the circulation can be carried on after the heart has ceased to act, the last act of that organ leaving the ventricles in the state of diastole.

If ever the arteries take on a state of alternate dilatation and contraction, corresponding with the pulse, we might expect it to happen when the volume of blood is reduced by great, and especially by sudden evacuations. Here the usual state of forced distention in which the arteries are found during health, being removed, they might yield to the distending force of the blood from the ventricle, which would then be propelled to the extremity of its course, by a series of successive contractions. The full, soft, undulating pulse, where wave seemed to succeed to wave, as well as the peculiar jerk met with in these cases, would give the idea of the vessel under the finger being alternately in a state of dilatation and contraction; yet this was not observed to be the case, by Dr. Parry, in two ewes, killed by repeated bleedings, where the diameter of the carotid is reported as progressively decreasing, until immediately before death, when it recovered again a little in one instance: but, to ascertain this point satisfactorily, it would be necessary to institute a more extended series of experiments. At all events, we may conclude, that no dilatation of the arteries can take place, until the usual relation between the power of the heart and the resistance of their coats has been destroyed, by giving a preponderance to the former; neither would this invalidate the general subserviency of the contractile powers of the arteries to the support of the circulation; and we thus acquire a more extended view of the resources of Nature, and of the wisdom of that provision, which has not contrasted the maintenance of the circulation to the powers of the heart alone, however greatly endowed, but has made every part of the vascular system conducive to the same great end.

C. E. Lucas, M. D.

Hatfield, May 18, 1820.

